# Pleuroparenchymal fibroelastosis: a rare interstitial lung disease

**DOI:** 10.1002/rcr2.108

**Published:** 2015-05-22

**Authors:** John C English, John R Mayo, Robert Levy, John Yee, Kevin O Leslie

**Affiliations:** 1 Department of Pathology and Laboratory Medicine, Vancouver General HospitalVancouver, British Columbia, Canada; 2 University of British ColumbiaVancouver, British Columbia, Canada; 3 Department of Radiology, Vancouver General HospitalVancouver, British Columbia, Canada; 4 Division of Respirology, Department of Medicine, Vancouver General HospitalVancouver, British Columbia, Canada; 5 Division of Thoracic Surgery, Department of Surgery, Vancouver General HospitalVancouver, British Columbia, Canada; 6 Department of Pathology, Mayo Clinic ArizonaScottsdale, Arizona; 7 Mayo Clinic Medical SchoolScottsdale, Arizona

**Keywords:** Pleuroparenchymal fibroelastosis (PPFE)

## Abstract

Pleuroparenchymal fibroelastosis (PPFE) is a newly described form of interstitial lung disease that originates in the upper lung zones and typically progresses to involve the entire lung. The disease may be idiopathic but is often associated with other pre- or coexisting conditions. Pneumothorax is a common complication and can occur at presentation or at other times during the course of the disease. Pathologically, interstitial fibrosis takes the form of a dense consolidation with some preservation of alveolar septal outlines and demonstrates a distinctly abrupt interface with residual normal lung. Unrecognized cases of PPFE may be incorrectly diagnosed as sarcoidosis, atypical idiopathic pulmonary fibrosis, or other unclassifiable interstitial pneumonias.

## Introduction

Pleuroparenchymal fibroelastosis (PPFE) is a recently recognized unusual form of fibrosing interstitial pneumonia with distinctive radiological and pathological characteristics. Upper lung zones are preferentially involved by a distinctive form of obliterative airspace fibrosis that is often associated with pneumothorax. Many cases are associated with underlying conditions but the majority are idiopathic and are formally recognized as one of the idiopathic interstitial pneumonias.

## Case Report

A 59-year-old woman underwent bilateral lung transplantation for interstitial lung disease following 25 years of progressive dyspnea, 33 months after referral to the lung transplantation clinic. The patient experienced recurrent respiratory tract infections and demonstrated abnormalities of the chest roentgenogram and had been treated for presumptive tuberculosis. Exacerbations occurred approximately three times a year with increasing dyspnea and sputum production, treated with antibiotics and corticosteroids. No specific pathogens were ever identified. The patient denied any relevant exposures, or substance use, and did not endorse any symptoms typical for the spectrum of connective tissue diseases. Past medical history included osteoporosis and gastrointestinal reflux. Current medications included oxygen therapy, azithromycin three times weekly, long-acting beta agonist/inhaled corticosteroid, ramipril, alendronate, and a proton pump inhibitor.

Examination revealed diminished breath sounds, bronchial breathing in the right lower lung zone, and crackles in the left base. On room air O_2_ saturation was 88%. There were no findings of pulmonary hypertension or clubbing.

Laboratory investigation was essentially within normal limits. Echocardiography showed mild-to-moderate mitral insufficiency, moderate tricuspid insufficiency, moderate diastolic dysfunction, and right ventricular enlargement, with normal function. Cardiac catheterization demonstrated normal coronaries, left ventricular ejection fraction 65%, and pulmonary arterial pressures 41/25/15 mmHg.

**Figure 1:**
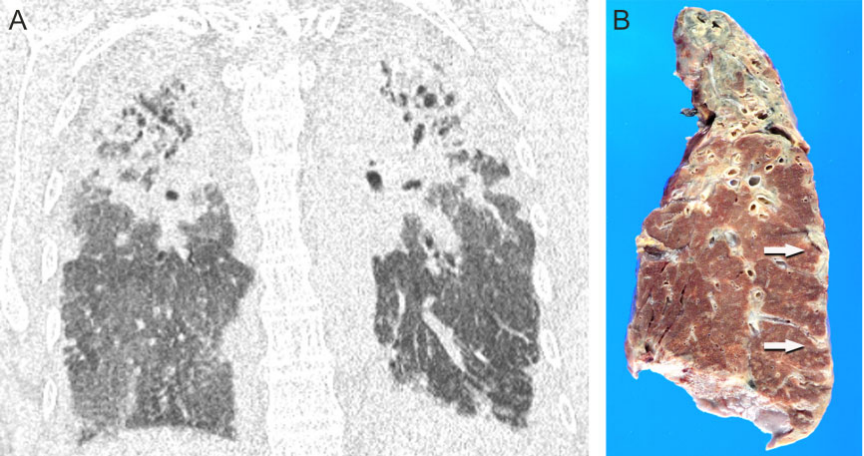
(A) Coronal reformatted computed tomography scan showing consolidative density effacing both upper lobes with pleural thickening noted extending to the lower zones. Cystic bronchiectasis is present. (B) Gross photograph of a coronal section of the left lung showing collapse and fibrosis of the entire upper lobe. Pleural fibrosis with nodular accentuation is noted in the lateral aspect (arrows).

High-resolution computed tomography scan (HRCT) imaging performed 7 years prior to lung transplantation showed bilateral cystic bronchiectasis in the upper lobes with associated volume loss, attended by pleural, subpleural, and perifissural fibrosis with nodular accentuation. Over time there was progression of the peribronchovascular and subpleural fibrosis with prominent cystic and varicoid bronchiectasis, all more pronounced in the upper lobes (Fig. 1A ). There were documented increased consolidative densities in the right upper lobe and progressive diminishment of lung volumes with focal air trapping.

**Figure 2:**
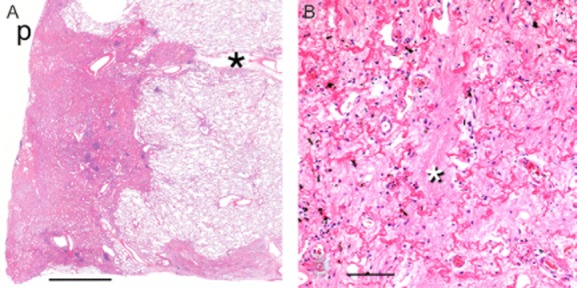
(A) Scanning micrograph demonstrating pleural and subpleural obliterative fibrosis and characteristic sharp interface with normal lung parenchyma. p, pleural surface; (*), interlobular septum. H&E stain. Bar = 5.0 mm. (B) Medium-power photomicrograph of an area of so-called obliterative fibrosis with prominent eosinophilic fragments of elastic lamellae roughly demarcating alveolar walls and intra-alveolar filling with old collagenous fibrotic plugs (*). Hematoxylin and eosin stain. Bar = 250 μm.

Pathological examination of the explanted lungs showed a distinctive band-like pattern of interstitial fibrosis, with subpleural and paraseptal distribution with near-total replacement of the upper lobes by fibrosis with traction bronchiectasis and volume loss, and progressively less disease tending toward the lung bases (Fig. 1B). Microscopic examination showed a consistent and distinctive pattern of preservation of the alveolar architectural outline by reduplicated elastic fibers enclosing airspaces obliterated by slightly looser connective tissue, all sharply demarcated from the residual normal parenchyma (Fig. 2A,B ). Foci of obliterative bronchiolitis and bronchiolectasis with mucostasis were noted. A few small non-necrotizing granulomas were encountered; one demonstrated fungal spores on the Grocott methenamine silver stain.

## Discussion

To date, approximately 100 cases of PPFE have been described in the literature. The defining features are radiographic pleuroparenchymal abnormalities, accentuated in the upper lobe, with corresponding histopathological changes of a distinctive fibroelastosis involving the peripheral/subpleural regions of the lung. Pneumothorax, spontaneous or iatrogenic, complicates the disease course in approximately 30% of cases. There are a high proportion of never smokers, with lesser numbers of ex- and current smokers. Clinical outcome in PPFE is variable with a significant number of patients demonstrating progressive decline and death. Survival characteristics depend on the stage of the disease at presentation. Aside from lung transplantation, there is no demonstrated effective treatment. Most cases are considered idiopathic although a variety of associated conditions have been described, including carcinoma treated with chemo- and/or radiotherapy [1], hematopoietic malignancies treated with chemotherapy and bone marrow transplantation [2], inhalatory exposures [3], infections [3], [4], gastrointestinal reflux disease [1], post-lung transplantation [5],[6], and in coexistence with usual interstitial pneumonia (UIP) [3].

HRCT studies of PPFE show subpleural reticular/nodular opacities initially favoring the upper zones [1] with pleural fibrosis and linear or wedge-shaped extensions down secondary lobular septa [3]. The mid- and lower zones are initially spared but are progressively enveloped with time [4]. Additional features include volume loss, traction bronchiectasis, and honeycomb remodeling that may signify coexistent UIP [3], centrilobular nodularity [1], mosaic attenuation [2], pneumothorax (uni- or bilateral) [2], and pneumomediastinum [4].

Radiological differential diagnoses may include chronic hypersensitivity pneumonitis, atypical usual interstitial pneumonitis, collagen vascular diseases, irradiation injury, asbestos exposure, prominent apical cap fibrosis, and sarcoidosis. Criteria for the radiological diagnosis of PPFE have been suggested [3].

The typical PPFE case, usually demonstrated on surgical biopsy, shows dense collagenous fibrosis with or without concomitant elastosis of the visceral pleura, pronounced in the apical region with progressive extension, often discontinuous, into the inferior zones. Dense subpleural bands of an obliterative parenchymal fibroelastosis, with vague preservation of alveolar outlines, follow this same distribution. The interface with uninvolved parenchyma is characteristically abrupt. Random intralobular or centrilobular nodules of fibroelastosis can be present [5]. Larger confluent or band-like areas of fibrosis can encase ectatic airways. Parenchyma away from the fibrosis is usually normal; however, small airway changes of obliterative bronchiolitis [2] with concomitant obstructive airspace exudates are identified in some cases [5]. Acute lung injury pathology, with possible thromboemboli or granulomas, has been reported in transplanted lungs with PPFE, as part of the spectrum of restrictive allograft syndrome [5],[6].

The precise pathophysiology of PPFE remains unknown although the common association with various forms of airway disease and a characteristic form of obliterative alveolar fibroelastosis suggests a form of incomplete resolution of acute or subacute lung injury, including atelectasis. The link with coexistent interstitial lung disease [3] requires further investigation.

## Disclosure Statements

No conflict of interest declared.

Appropriate written informed consent was obtained for publication of this case report and accompanying images.
